# Developing a professionalism curriculum on the nonmedical use of prescription stimulants among medical students

**DOI:** 10.5116/ijme.5d9b.1565

**Published:** 2019-10-25

**Authors:** Suranjana Dey, Gabrielle Lindley, Elizabeth Ma, Caroline Harada, Mariko Nakano-Okuno

**Affiliations:** 1 School of Medicine, the University of Alabama at Birmingham, Birmingham, Alabama, USA; 2 Psychiatry Residency Program, Loma Linda University, Loma Linda, California, USA; 3 Department of Medical Education, School of Medicine, the University of Alabama at Birmingham, Birmingham, Alabama, USA

**Keywords:** Professionalism curriculum, nonmedical use of prescription stimulants, medical students

## To the Editor

Prescription stimulants are medications commonly prescribed to treat conditions such as attention-deficit hyperactivity disorder (ADHD) and narcolepsy. In the United States, these medications include amphetamines and methylphenidate, both of which have a high potential for abuse that may lead to severe psychological or physical dependence. However, these stimulants are often used by college students^1^^,^^2^ and even medical students^3^^,^^4^^,^^5^^,^^6^ for nonmedical purposes, such as to stay awake before taking an exam or for recreational use. This problem has been accompanied by numerous calls to action from those within the medical education community,^7^^,^^8^^,^^9^^,^^10^  and there is general agreement that  non-prescribed stimulant (NPS) use among medical students not only represents an ongoing crisis in students’ academic integrity and general well-being,^7^^,^^11^ but may also modify future physicians’ beliefs and conduct as they relate to professionalism and substance use.^12^^,^^13^  Though experts suggest the importance of designing effective interventions to help curb NPS use in medical students, there is currently limited data on successful strategies to this end. Here, we describe the development of an ethics/professionalism-focused educational curriculum on NPS use for the medical students at our institution, in an effort to decrease the prevalence of NPS use.

Prior to developing our curriculum, a needs assessment was conducted, with approval from the institutional review board of the University of Alabama at Birmingham, in which 186 second-year medical students were asked to complete a survey. The survey was completed by 59 students (response rate: 31.7%), showing that 11.8% of the surveyed students had engaged in NPS use at some point in their life and 20.3% had been prescribed a stimulant for a diagnosed medical condition.  Although the majority (79.7%) of the respondents did not think NPS use is ethically justifiable, 16.9% thought NPS use could be ethically justified, and 6.8% reported that they would accept a stimulant that was not prescribed to them if offered by a classmate.

Based on this needs assessment, we designed a 45-minute educational session that was administered to all second-year medical students at our medical school in the 2017-2018 academic year.  The session was held at a time of year when students were also learning about the neurophysiology of ADHD and of substance dependence during their neurosciences pre-clinical module. The session was incorporated into a pre-existing course focusing on ethics, professionalism, and wellness among medical students, called Learning Communities (LC).  It was a small group discussion facilitated by a faculty member who serves as the LC mentor. Students first watched a video summarizing data from a student survey on NPS use at our institution.  They then received a handout covering basic background information on the topic of NPS use drawn from recent literature, relevant news coverage, and expert sources.  The handout included information on resources for those seeking further information about NPS use or withdrawal.

Students were then asked to discuss whether NPS use could be ethically justified and whether NPS use by medical professionals contradicts physicians’ professional duty for ethical behavior.   Discussion questions included: “Is NPS a form of cheating?”, “Will it foster a culture of dependency on medication for schooling?”, “How is it different from caffeine intake?”, “Do you think there are cases in which medical professionals should be allowed to take stimulants to accomplish their job – surgeons or emergency doctors who must stay awake overnight, for example?”, and “Is it ever justifiable for a medical professional to take prescription stimulants himself/herself for non-medical purposes? Why or why not?”.

Feedback from students was largely encouraging while suggestive of room for improvement. According to the mid-year course evaluations completed by 167 second-year students (response rate: 89.8%), 72.5% of respondents reported that they either “agreed” or “strongly agreed” that the “Ethics of Stimulant Use in the Professional Setting” session was effective.  To obtain more detailed information on student reactions to the course, a second survey was administered (with approval from the UAB IRB).  The response rate was 25.3% (47 out of 186 students), showing 46.8% of respondents “agreed” or “strongly agreed” that their knowledge of the arguments for and against NPS use increased.  Additionally, 8.5% of respondents agreed that their opinions about NPS use had changed as a result of the session, although the survey did not allow us to determine in which direction their opinion had changed.  Even after the session, 12.8% of respondents responded that NPS use was ethically justifiable, and 19% felt that there are cases in which medical students should be allowed to take prescription stimulants for non-medical purposes in order to accomplish their academic tasks, although the percentage of respondents who answered that they would “accept a stimulant that was not prescribed to them if offered by a classmate” was only 4.2%.

To our knowledge, this is the first report of an attempt to develop an ethics/professionalism curriculum focused on NPS use among medical students.  We were able to demonstrate that building such a curriculum is feasible and that it was favorably perceived by students. It is difficult to interpret the results of our follow up survey due to low response rates and lack of a “pre/post” survey design, but it does appear that, even after the educational session, there are students who believe NPS use is ethically justifiable.  It may be that these numbers were even higher prior to the session, given the fact that some respondents did report that the session changed their minds.  We were disappointed in these results and have considered whether it was related to issues of timing, content, or format.  We intend to move the session to the first year of the curriculum instead of the second year, given the fact that our needs assessment demonstrated that some students report they are already exposed to NPS or medical stimulant use by the second year of medical school. It is our hope that by intervening earlier in the curriculum, perhaps we can deter medical students from trying NPS in the first place. Further review of the content and format will be necessary to better understand why so many students still felt NPS use was justified. It is possible that different content or a longer, more interactive format might have a greater impact on students. Limitations to this project include the low response rate to our follow up survey and the lack of a pre/post survey to determine change in knowledge and attitudes.  Further study is needed to assess if and how educational attempts such as ours can change medical students’ beliefs and/or decision-making regarding NPS use. Our attempt to develop an educational session on NPS use should be viewed as the first step toward building a robust curriculum to address the challenge of NPS use among medical students, a major concern in the development of a physician’s professional identity.

### Conflict of Interest

The authors declare that they have no conflict of interest.
